# Testing sampling bias in estimates of adolescent social competence and behavioral control

**DOI:** 10.1016/j.dcn.2020.100872

**Published:** 2020-10-22

**Authors:** M. Fakkel, M. Peeters, P. Lugtig, M.A.J. Zondervan-Zwijnenburg, E. Blok, T. White, M. van der Meulen, S.T. Kevenaar, G. Willemsen, M. Bartels, D.I. Boomsma, H. Schmengler, S. Branje, W.A.M. Vollebergh

**Affiliations:** a Utrecht University, Utrecht, The Netherlands; b Erasmus Universiteit, Rotterdam, The Netherlands; c Leiden University, The Netherlands; d Vrije Universiteit Amsterdam, Amsterdam, The Netherlands; e University of Groningen, University Medical Center Groningen, Groningen, The Netherlands

**Keywords:** Socioeconomic status, Adolescence, Social competence, Behavioral control, Selection bias

## Abstract

In 5 of the 6 large Dutch developmental cohorts investigated here, lower SES adolescents are underrepresented and higher SES adolescents overrepresented. With former studies clearly revealing differences between SES strata in adolescent social competence and behavioral control, this misrepresentation may contribute to an overestimation of normative adolescent competence. Using a raking procedure, we used national census statistics to weigh the cohorts to be more representative of the Dutch population. Contrary to our expectations, in all cohorts, little to no differences between SES strata were found in the two outcomes. Accordingly, no differences between weighted and unweighted mean scores were observed across all cohorts. Furthermore, no clear change in correlations between social competence and behavioral control was found. These findings are most probably explained by the fact that measures of SES in the samples were quite limited, and the low SES participants in the cohorts could not be considered as representative of the low SES groups in the general population. Developmental outcomes associated with SES may be affected by a raking procedure in other cohorts that have a sufficient number and sufficient variation of low SES adolescents.

## 1 Introduction

Although cohort studies generally aim at selecting a sample that is representative for the whole population, vulnerable groups in our society are less often part of these cohort studies (Jang and Vorderstrasse, 2019; Svensson et al., 2012; Walter et al., 2013). Since participants from a lower socioeconomic background tend to be less inclined to participate in research, this can result in a sampling bias of participants with a higher socioeconomic status (SES; Bornstein et al., 2013; LeWinn et al., 2017). An important question that follows is whether findings from such samples reflect the psychosocial development of the whole population or of a subsample of our society (Arnett, 2008; Boudewijns et al., 2019; Henrich et al., 2010; LeWinn et al., 2017). One way to answer this question is to estimate to what extent the unweighted results of such samples diverge from the results when samples are weighed with respect to SES. In the current study, we investigate whether estimates of social competence and behavioral control in adolescents from 6 Dutch developmental cohorts differs between the unweighted samples and their weighted samples that are more socioeconomically representative of the general Dutch population.

### 1.1 SES, social competence, and behavioral control

Several studies underline the positive association between family SES and an adolescent’s social competence (e.g., de Laat et al., 2016; Hosokawa and Katsura, 2017) and behavioral control (e.g., Bradley and Corwyn, 2002; Farley and Kim-Spoon, 2017). A family’s SES reflects the relative position of the household in the wealth distribution of a given society (De Neubourg et al., 2018). While no single definition of SES is universally accepted, it is generally measured through a combination of family income, parental education, and parental occupation (Krieger et al., 1997; Oakes and Rossi, 2003) to approximate an individual’s resources, prestige, knowledge and power (Link-Gelles et al., 2016). Given that SES indicators may differ in stability across time and in predicting adolescent development (Duncan & Magnuson, 2003), SES is preferably estimated using multiple indicators instead of a single indicator (Green and Popham, 2019; Thaning and Hällsten, 2020). Social competence refers to an individual’s ability to engage in meaningful interactions with peers and adults (Fabes et al., 2006; Rose-Krasnor, 1997). Behavioral control refers to the ability to control one’s behaviors, cognitions, and emotions and to adapt to rules. It is often termed as self‐regulation, effortful control, or self-control in the literature (Nigg, 2017; Zhou et al., 2012).

The interactionist model (Conger and Donnellan, 2007; Martin et al., 2010) postulates that high family SES positively impacts psychosocial development in children and adolescents. Economic hardships – often accompanying low SES families – cause prolonged stress in parents which interferes with effective child rearing practices (i.e., family stress model); while material resources – more easily invested by high SES families – can stimulate psychosocial development (i.e., family investment model). Though distinct characteristics, social competence and behavioral control tend to develop interactively (Cunha and Heckman, 2007), with more socially competent adolescents generally also displaying better behavioral control, and vice versa. For example, adolescents with better self-regulatory capacities are less likely to engage in transgressive behaviors and more likely to engage in prosocial behaviors towards others (Farley and Kim-Spoon, 2014; Meldrum and Hay, 2012).

### 1.2 SES and research participation

In developmental cohort studies, low SES participants may be undersampled or underrepresented. Low SES participants are undersampled if the proportion of low SES participants in the sample is smaller than the proportion high SES participants in the sample. Low SES participants are underrepresented if the proportion of low SES participants in the sample is smaller than the proportion low SES participants in the target population (see Skiba et al., 2008). Though in both cases the sample has too few participants from a low SES background, undersampling and underrepresentation yield different research challenges.

Undersampling of low SES participants has a direct, negative impact on a study’s power to detect effect sizes (Bornstein et al., 2013; Button et al., 2013). The absolute number of low SES participants would limit the range and complexity of research questions in which developmental differences across socioeconomic strata can be investigated (given that higher SES participants are more likely to participate). Underrepresentation of low SES participants is problematic for understanding normative psychosocial development in a given target population (Brady et al., 2018; LeWinn et al., 2017). A fundamental goal of developmental research is to distinguish universal aspects of development from variable aspects of development, caused by – for example – socioeconomic status (Brady et al., 2018). This requires studying a representative sample of the population. Cohort studies have substantially contributed to our understanding of adolescent’s psychosocial development, even though they encounter many challenges at the stages of participant recruitment. However, as a result of the common underrepresentation of low SES adolescents in cohort studies, normative psychosocial development may be overestimated when extrapolating research findings to the general population. The usual way to correct for these biases is by weighing the sample data to the population on variables for which the population distribution is known.

This challenge of selection bias is increasingly being recognized in other, related fields of research (e.g., Falk et al., 2013; Paus, 2010). For example, after a predominantly high SES child and adolescent cohort was weighted to national SES statistics, an attenuation in normative neurological growth was observed (LeWinn et al., 2017). Given the strong association between brain development and psychosocial development, normative social competence and behavioral control may similarly be overestimated in adolescents if we rely primarily on high SES samples. In related genetics research, population heritability estimates of for example cognitive ability (Gottschling et al., 2019; Turkheimer et al., 2003) were attenuated after heritability estimates were found to be lower in samples of low SES children and adolescents compared to previously studied high SES samples. In recent years, participation rates in community research have been dropping steeply (Galea and Tracy, 2007; Nohr and Liew, 2018), especially among more vulnerable groups resulting in an even stronger reliance on predominantly higher SES research samples. However, it must also be noted that some normative estimates of development which are based on high SES samples are similar to estimates in the socioeconomically diverse population (see for example Pizzi et al., 2012). Though these examples are seemingly unrelated to adolescent social competence and behavioral control, it suggests that a similar attenuation of normative estimates may be observed when weighing our developmental cohorts to population SES statistics.

We aim to extend the existing progress in this field by investigating the possible influence of an underrepresentation of low SES adolescents on normative social competence and behavioral control. All 6 Dutch developmental cohorts studied here are part of the Consortium on Individual Development (CID). CID aims to examine how environmental (e.g., SES) and individual (e.g., genetic makeup) characteristics influence the development of social competence and behavioral control; skills that are essential for functioning in society and reducing risk of behavioral and emotional problems.

First, the cohorts were evaluated for socioeconomic representativeness; second, we assessed the impact of SES on social competence and behavioral control, and the effect of deviations in representativeness on estimates of adolescent social competence and behavioral control. By comparing weighted estimates of adolescent social competence and behavioral control to unweighted estimates, our aim was to quantify the effect of a possible sampling bias on normative adolescent psychosocial competence. Additionally, we explored the effect of sample weighing on the association between adolescent social competence and behavioral control.

## 2 Methods

### 2.1 Participants

Participants of 6 large cohort studies from The Netherlands were investigated: Generation R (GenR), Leiden Consortium on Individual Development (L-CID), Research on Adolescent Development And Relationships (RADAR), the Netherlands Twin Register (NTR), Tracking Adolescents’ Individual Lives Survey (TRAILS), and YOUth (Youth of Utrecht) (Table 1). *Generation R* is a birth cohort study from the Rotterdam municipality, an urban region in the west of the Netherlands (Kooijman et al., 2016). Measurements relevant for this study were collected from 9−12-year-old children, who have been participants since before birth. *L-CID* is a longitudinal experimental twin-study, which aims to study the effect of a video-intervention on parental sensitivity and sensitive discipline in two twin cohorts (early childhood and middle childhood) (Crone et al., 2019; Euser et al., 2016). Families with same sex twins were recruited from the western region of the Netherlands. The l-CID participants in our study were 9−10-year-old children of age from the middle childhood cohort, who had been allocated to the control group and did not receive an intervention. *NTR* is a longitudinal twin study that aims to identify genetic and environmental factors of behavioral and emotional problems in children and adolescents (Bartels et al., 2007). Participants are recruited across the entire country, and research assessments are attuned to individual age. For the current study, data collected between 2003 and 2017 of 10-year-olds was analyzed. Though earlier data is available in NTR (i.e., since 1987), high quality national census statistics were scarce before 2003. Also, this time period restriction matches well with the time periods of the other cohorts in this study. *RADAR* is a longitudinal cohort study that investigates interactions and conflicts of adolescents with parents and peers, emotional development, identity, and internalizing and externalizing problem behavior (Van Lier et al., 2008). Participants have been recruited through elementary schools in the Utrecht municipality (i.e., mid-Netherlands) and 4 large cities elsewhere. Baseline measurements were used in this study, at which adolescents were around age 13. *TRAILS* is a general population cohort study that aims to understand (the interaction between) determinants of mental health and social development during adolescence and young adulthood (Huisman et al., 2008; Oldehinkel et al., 2015). Participants are recruited from urban and rural areas in the northern region of the Netherlands. Baseline measurements were used in this study, at which adolescents were approximately age 13−14. *YOUth* is a longitudinal cohort study following two separate groups of participants from the Utrecht region (i.e., mid-Netherlands) either in their development from pregnancy into childhood or from childhood into adolescence (in this special issue: Onland-Moret, Kemner, & Hulshoff Pol, 2020 (under submission)). The 9-year old children whose data were analyzed for this study were recruited through elementary schools, municipal health services, and local neighborhood centers.

**Table 1:** Demographics of participants from the 6 cohorts.

Cohort	Wave	*n*	Age (SD)	Female	Dutch a
*GenR*	T4	3895	9.7 (0.28)	50.1 %	76.5 %
*L-CID*	T3	142	9.5 (0.64)	52.8 %	100.0 %*
*NTR*	T5	6266	9.9 (0.54)	50.3 %	94.5 %
*RADAR*	T1	441	13.0 (0.44)	44.2 %	93.3 %*
*TRAILS*	T2	1535	13.5 (0.52)	50.4 %	89.8 %
*YOUth*	T1	595	9.5 (0.87)	54.5 %	95.6 %

a or Western European origin.

* Dutch or Western European mother and father as inclusion criterion.

We aimed to align the mean ages between cohorts, ranging from late childhood to early adolescence (9.5–13.5 years old). Participants from all cohorts were predominantly of Dutch or Western European origin (see Table 1). Multiple siblings participated in Generation R, NTR, RADAR, and l-CID. For Generation R and NTR, one adolescent per family was randomly selected to be retained for analyses. In RADAR, the targeted adolescent and not the consulted sibling was retained per family for analyses. In l-CID, the adolescent who had been randomly allocated to the control group and did not receive an intervention was retained for analyses.

Besides cohort specific inclusion and exclusion criteria, our analytical approach required participants to i) have observations on all SES variables used for weighing, and ii) have at least one observed score, for social competence or behavioral control.

### 2.2 Materials

All cohorts have collected data on adolescents’ SES, social competence and behavioral control. Although none of the measuring instruments has been administered consistently across all cohorts, considerable overlap can be observed. Measures of social competence and behavioral control were selected to facilitate cross-validation across cohorts and age ranges (i.e., assessing the same measurement instrument in multiple cohorts when possible; see also Table 7).

#### 2.2.1 Socioeconomic status

SES was measured with mother’s educational attainment, father’s educational attainment, and family income. Highest level of educational attainment of both mother and father was identified in all six cohorts. For our analyses, three ordinal levels of educational attainment were constructed in the cohorts and the census: lower education, middle education, and higher education. Lower education includes for example primary school as highest attained level of education; middle education includes for example tertiary vocational education; and higher education includes for example university education (see Appendix A for more detail about the Dutch education system and our classification approach).

Family income has been collected in Generation R, TRAILS, and YOUth; but not in l-CID, RADAR, and NTR. In Generation R and TRAILS, net family income per month was obtained from the primary participating parent. In YOUth, gross family income per month was obtained from both parents. In case of discrepancy (*n* = 145; 17.3 %), the answer of the primary participating parent was leading. For these cohorts, income categories were matched to income deciles from the national census (for full procedure, see Appendix B).

Across cohorts, Pearson’s correlations of mother’s education and father’s education ranged between *r* = 0.31 to *r* = 0.54, of mother’s education and income between *r* = 0.24 to *r* = 0.44, and of father’s education and income between *r* = 0.29 to *r* = 0.52 (see Appendix C).

Per cohort, we used the census distributions of observed SES variables (Appendix D) corresponding to the starting year of data collection of men and women between age 35 and 55 (Table 2, Table 3, Table 4, Table 5, 8, 9). For NTR, in which inclusion is ongoing and data collection waves are individually based on participant’s age, census statistics between 2003 and 2017 were averaged to indicate Dutch population SES.

**Table 2:** Generation R versus Dutch population (2008/2012)^1^. Mean item scores (SD) and Pearson’s correlations (r) on social competence and behavioral control per SES variable category in unweighted sample.

	Dutch population	**GenR**	Δ	:	Social competence 2	Behavioral control 3	r
Mother education (%)							
Lower education	34.4	1.4	−33.0	0.04	1.78 (0.21)^a^	1.65 (0.41)^b^	0.65
Middle education	40.7	32.9	−7.8	0.81	1.84 (0.20)^a^	1.62 (0.36)^a^	0.61
Higher education	24.9	65.7	40.8	2.64	1.86 (0.19)^b^	1.66 (0.35)^b^	0.58
Father education (%)							
Lower education	26.8	3.9	−22.9	0.15	1.83 (0.22)^a^	1.65 (0.37)^b^	0.75
Middle education	41.1	33.8	−7.3	0.82	1.83 (0.21)^a^	1.60 (0.37)^a^	0.62
Higher education	32.1	62.2	30.1	1.94	1.86 (0.18)^b^	1.67 (0.34)^b^	0.56
Net family income (%)							
< €1.200	14.5	4.0	−10.5	0.28	1.78 (0.24)^a^	1.60 (0.39)^a^	0.72
€1.200 – 2.000	24.0	10.4	−13.6	0.43	1.83 (0.23)^ab^	1.58 (0.40)^a^	0.65
€2.000 – 3.200	29.0	14.9	−14.1	0.51	1.83 (0.20)^ab^	1.62 (0.37)^a^	0.62
€3.200 – 4.000	13.0	19.4	6.4	1.49	1.85 (0.18)^b^	1.63 (0.36)^a^	0.52
> €4.000	19.5	51.4	31.9	2.64	1.87 (0.18)^c^	1.68 (0.33)^b^	0.57

Parameters with different superscripts differ significantly from each other at the *p* < .05 level.

Δ = difference in percent points between GenR and Dutch population = GenR – Dutch population.

: = ratio of GenR to Dutch population = GenR ÷ Dutch population.

1 Educational attainment from 2008 census; Family income from 2012 census.

2 CBCL-SP.

3 ASCS.

**Table 3:** NTR versus Dutch population (2003-2017). Mean item scores (SD) and Pearson’s correlations (r) of social competence and behavioral control per SES variable category in unweighted sample.

	Dutch population	**NTR**	*Δ*	:	Social competence 1	Behavioral control 2	*r*
*Mother education (%)*							
Lower education	26.7	30.8	4.1	1.15	1.82 (0.21)^a^	1.56 (0.38)^a^	0.65
Middle education	44.3	35.0	−9.3	0.79	1.83 (0.20)^a^	1.61 (0.35)^b^	0.63
Higher education	29.0	34.2	5.2	1.18	1.85 (0.18)^b^	1.67 (0.32)^c^	0.61
*Father education (%)*							
Lower education	23.6	31.8	8.2	1.35	1.82 (0.21)^a^	1.57 (0.38)^a^	0.65
Middle education	42.7	30.8	−11.9	0.72	1.83 (0.20)^a^	1.61 (0.35)^b^	0.63
Higher education	33.7	37.4	3.7	1.11	1.85 (0.19)^b^	1.66 (0.32)^c^	0.62

Parameters with different superscripts differ significantly from each other at the *p* < .05 level.

Δ = difference in percent points between NTR and Dutch population = NTR – Dutch population.

: = ratio of NTR to Dutch population = NTR ÷ Dutch population.

1 CBCL-SP.

2 ASCS.

**Table 4:** TRAILS versus Dutch population (2001/2003)^1^. Mean item scores (SD) and Pearson’s correlations (r) on social competence and behavioral control per SES variable category in unweighted sample.

	Dutch population	**TRAILS**	*Δ*	:	Social competence 2	Behavioral control 3	*r*
*Mother education (%)*							
Lower education	37.2	34.1	−3.1	0.92	1.82 (0.22)^a^	1.52 (0.39)^a^	0.55
Middle education	40.3	36.2	−4.1	0.90	1.83 (0.23)^ab^	1.55 (0.38)^a^	0.58
Higher education	22.5	29.8	7.3	1.32	1.86 (0.18)^b^	1.62 (0.36)^b^	0.48
*Father education (%)*							
Lower education	29.3	30.8	−1.5	1.05	1.83 (0.20)^ab^	1.52 (0.39)^a^	0.59
Middle education	41.2	32.2	−9.0	0.78	1.82 (0.24)^a^	1.52 (0.40)^a^	0.52
Higher education	29.4	36.9	7.5	1.26	1.86 (0.19)^b^	1.63 (0.34)^b^	0.53
*Net family income (%)*							
< €1.135	22.2	7.0	−15.2	0.32	1.80 (0.23)^ab^	1.52 (0.39)^ab^	0.65
€1.135 – €1.590	15.7	16.9	1.2	1.08	1.79 (0.24)^a^	1.52 (0.37)^a^	0.46
€1.590 – €2.045	19.0	23.7	4.7	1.25	1.84 (0.20)^ab^	1.54 (0.36)^ab^	0.58
€2.045 – €2.955	23.2	35.1	11.9	1.51	1.84 (0.22)^b^	1.58 (0.40)^ab^	0.57
> €2.955	19.9	17.3	−2.6	0.87	1.88 (0.19)^b^	1.62 (0.35)^b^	0.49

Parameters with different superscripts differ significantly from each other at the *p* < .05 level.

Δ = difference in percent points between TRAILS and Dutch population = TRAILS – Dutch population.

: = ratio of TRAILS to Dutch population = TRAILS ÷ Dutch population.

1 Family income from 2001 census; Educational attainment from 2003 census.

2 CBCL-SP.

3 ASCS.

**Table 5:** YOUth versus Dutch population (2015). Mean item scores (SD) and Pearson’s correlations (r) on social competence and behavioral control per SES variable category in unweighted sample.

	Dutch population	**YOUth**	*Δ*	:	Social competence a	Behavioral control b	*r*
*Mother education (%)*							
Lower education	22.3	3.0	−19.3	0.13	1.68 (0.37)	1.47 (0.30)	0.19
Middle education	43.4	22.0	−21.4	0.51	1.74 (0.34)	1.45 (0.42)	0.14
Higher education	34.3	75.0	40.7	2.19	1.69 (0.34)	1.53 (0.39)	0.32
*Father education (%)*							
Lower education	22.0	7.1	−14.9	0.32	1.75 (0.31)	1.45 (0.35)	0.21
Middle education	42.2	24.0	−18.2	0.57	1.71 (0.33)	1.52 (0.39)	0.26
Higher education	35.8	68.9	33.1	1.92	1.70 (0.35)	1.51 (0.40)	0.28
*Net family income (%)*							
< €1.250	9.0	1.5	−7.5	0.17	1.75 (0.49)	1.39 (0.29)	0.24
€1.250 – €2.000	13.8	4.0	−9.8	0.29	1.65 (0.39)	1.62 (0.32)	0.07
€2.000 – €3.000	16.6	6.4	−10.3	0.38	1.73 (0.28)	1.40 (0.47)	0.38
€3.000 – €4.000	13.0	15.8	2.9	1.22	1.65 (0.35)	1.42 (0.42)	0.34
> €4.000	47.6	72.3	24.9	1.52	1.72 (0.34)	1.53 (0.38)	0.26

Parameters with different superscripts differ significantly from each other at the *p* < .05 level.

Δ = difference in percent points between YOUth and Dutch population = YOUth – Dutch population.

: = ratio of YOUth to Dutch population = YOUth ÷ Dutch population.

a SDQ-PB.

b ASCS.

#### 2.2.2 Social competence

Social competence was operationalized in terms of social problems or prosocial behavior. In GenR, NTR, and TRAILS, parents reported on their adolescent’s social problems using the Social Problems-subscale from the Child Behavior Checklist (CBCL-SP; Achenbach, 1991; Achenbach and Rescorla, 2001). The CBCL-SP consists of 11 items (e.g, “Doesn’t get along with other boys and girls”) which either mother or father rated as 0 (Not true), 1 (Somewhat or sometimes true), or 2 (Very true or often true). After recoding, higher scores on the CBCL-SP indicate better social competence. With Cronbach’s alpha coefficients ranging from 0.72 to 0.76 across cohorts, the internal consistency of the CBCL-SP is adequate.

In l-CID and YOUth, prosocial behavior was measured with the Prosocial Behavior-subscale from the Strengths and Difficulties Questionnaire (SDQ; Goodman, 1997; Stone et al., 2010). The SDQ-PB consists of 5 items (e.g., “Often volunteers to help others”), rated as 0 (Not true), 1 (Somewhat true), or 2 (Certainly true), by either mother or father (YOUth) or both parents combined (L-CID). Higher scores on the SDQ-PB indicate better social competence. The internal consistency of the SDQ-PB is adequate, with a Cronbach’s alpha of 0.78 in l-CID and 0.72 in YOUth.

In RADAR, adolescents rated themselves on a cohort specific Prosocial Behavior questionnaire (R-PB), consisting of 11 items (e.g., “I’m normally kind to others”) on a scale from 1 (Totally not true) to 7 (Totally true). Higher scores on the R-PB indicate better social competence. For all instruments, participants’ mean item scores were calculated as measure of adolescent social competence. In YOUth, both the CBCL-SP and SDQ-PB were obtained: an interscale correlation of *r* = 0.29, *n* = 373, *p* < .001 indicated low convergent validity. For YOUth, the SDQ-PB was reported as measure of social competence to enable cross-validation of the SDQ-PB across two cohorts (i.e., l-CID). The Cronbach’s alpha coefficient of the R-PB is 0.89, suggesting good internal consistency.

#### 2.2.3 Behavioral control

Behavioral control was operationalized in terms of self-control or drive.

In Gen-R, NTR, and YOUth, self-control was measured with the parent-reported Achenbach Self-Control Scale based on CBCL items (ASCS; Willems et al., 2018). The ASCS consists of 8 items (e.g, “Impulsive or acts without thinking”) which either mother or father rated as 0 (Not true), 1 (Somewhat or sometimes true), or 2 (Very true or often true). After recoding, higher scores on the ASCS indicate better behavioral control. Across cohorts, the Cronbach’s alpha coefficients range between 0.81 and 0.83, suggesting good internal consistency.

In l-CID, RADAR, and TRAILS, we proposed to measure behavioral control using the BAS-Drive subscale from the Behavioral Inhibition System/Behavioral Activation System (BIS/BAS) questionnaire (BAS-D; Carver and White, 1994). Drive refers to stronger impulsivity or stronger positive affective reactions to signals of impending reward (e.g., Jiang and Zhao, 2017; Taubitz et al., 2015), and is associated with – but not synonymous to – poorer behavioral control. The BAS-D consists of 4 items (e.g., “When I want something, I usually go all-out to get it”), which adolescents self-report on a scale from 1 (Disagree strongly) to 4 (Agree strongly). After recoding, a higher score on the BAS-D indicates better behavioral control. Across cohorts, the Cronbach’s alpha coefficients range between 0.57 and 0.65, which is on the lower bound of acceptable reliability.

Following serious concerns about the validity of the BAS-D as a measure of behavioral control (e.g., poor convergent validity between the ASCS and BAS-D in TRAILS; *r* = 0.13, *n* = 1088, *p* < .001) but after having already completed our preregistration, we present these specific findings separately in the Supplementary Materials section at the end of this article (Tables 8–10). In TRAILS, both the ASCS and BAS-D were obtained, but only the use of BAS-D scores was preregistered. Hence, our findings in TRAILS that were based on non-preregistered ASCS data (Table 4) may be interpreted as exploratory.

### 2.3 Raking procedure

To understand how sample composition influences our understanding of adolescent social competence and behavioral control, we contrasted the unweighted versus the weighted sample of 6 large cohort studies from the Netherlands. The unweighted sample consisted of adolescents with complete observations on SES variables, and at least one observed score on social competence or behavioral control. The weighted sample was created using a raking procedure, and is representative of the Dutch population in terms of socioeconomic status. The unweighted sample and the weighted sample consisted of the exact same participants. National census data on parental education and income was retrieved from the open data portal of Statistics Netherlands (CBS Statline; see Appendix D).

Raking is a survey method through which weights are applied to individual participants based on census totals, so that the weighted sample better reflects the population distribution of SES variables that are included in the weighing procedure (Kalton and Flores-Cervantes, 2003; Kolenikov, 2014). With raking, the distribution of the sample is fitted to population values one variable at a time. Weights are fitted iteratively across all variables used in the weighting, and then re-weighted until the weight factors do not change much and ‘coverage’. After this, each participant is then assigned a final weight that will balance the sample distribution to the population distribution as well as possible for all variables in the model.

### 2.4 Mean differences and correlation differences

After raking, estimates of adolescent social competence and behavioral control were compared between unweighted and weighted samples. The difference in mean social competence and mean behavioral control between unweighted and weighted samples is expressed as effect size Cohen’s *d*. Standard interpretations of Cohen’s *d* apply, with *d* = 0.2 indicating a small effect; *d* = 0.5 a medium effect; and *d* = 0.8 a large effect of sample composition on estimates of adolescent social competence and behavioral control (Rosnow and Rosenthal, 2003).

In both the unweighted and weighted samples, we calculated the Pearson’s correlation coefficient *r* between social competence and behavioral control. This difference score can directly be interpreted as an effect size (Rosnow and Rosenthal, 2003).

### 2.5 Sensitivity analyses

Per cohort, we conducted a sensitivity analysis in which outliers were deleted. Differences between excluded and included adolescents per cohort are described in Appendix F. By excluding outliers on social competence or behavioral control, we aimed to control for the possibility of an overreliance on one or few observations in determining the weighted estimates. Scores with ≥ (±) 2.58 SD’s from the mean were considered as outliers. After consultation, this cut-off was considered more valid for rigid sensitivity testing than our preregistered cut-off of ≥ (±) 3 SD’s. For our two non-normally distributed outcome variables this corresponded to the exclusion of between 2.0%–4.1% of extreme scores across cohorts. If a participant’s score on either social competence or on behavioral control was an outlier, the participant was excluded from the raking procedure. Besides this preregistered sensitivity analysis, in all cohorts, we also reran our raking procedure with only 1 or 2 SES variables instead of all available SES variables.

## 3 Results

### 3.1 Cohort representativeness

As a first step, we compared the SES distribution of the 6 cohorts to Dutch population statistics in order to obtain weights for the raking procedure (Table 2, Table 3, Table 4, Table 5, 8, 9). For each cohort, we calculated the difference (i.e., *Δ* = cohort proportion – population proportion; expressed in percent points) in prevalence between Dutch population and cohort participants per SES variable category. Positive percentages indicate an overrepresentation of participants from this SES variable category, and negative percentages indicate an underrepresentation of participants from this SES variable category. We also calculated the ratio (: = cohort proportion ÷ population proportion) of underrepresentation (if : < 1) or overrepresentation (if : > 1) per SES variable category in each cohort. No rule of thumb is known for assessing representativeness, but sample deviations larger than 10 % from the population proportion have previously been considered as a warning sign (Chinn & Hughes, 1987 in Skiba et al., 2008). This corresponds to ratios between 0.9 and 1.1 being considered reasonable (e.g., if 30 % of mothers in the population are higher educated, 27–33 % of the sample should consist of higher educated mothers).

In 5 of the 6 cohorts, low SES participants are underrepresented and high SES participants are overrepresented (Table 2, Table 3, Table 4, Table 5, 8, 9). Over these 5 cohorts, the mean Δ = -13.7 % and the mean : = 0.43 for all low SES indicators. This is equivalent to if 25 % of Dutch adolescents would be of low SES, and the 5 cohorts on average consist of 11 % low SES adolescents. Similarly, over these 5 cohorts, the mean Δ = 23.6 % and the mean : = 1.78 for all high SES indicators. In other words, if 29 % of Dutch adolescents would be of high SES, these 5 cohorts would on average consist of 52 % high SES adolescents. The exception is NTR, with a modest overrepresentation of low SES and high SES adolescents, and slight underrepresentation of middle SES adolescents. These deviations in socioeconomic representativeness in all cohorts form an important first prerequisite for performing the raking procedure.

As expected, *F*-tests indicated differences in social competence and behavioral control between adolescents from different SES variable categories in Generation R (Table 2), NTR (Table 3) and TRAILS (Table 4). Post-hoc analyses indicated that in most of these cases, adolescents with lower educated parents from lower income families scored lower on social competence or behavioral control than adolescents with higher educated parents from higher income families; but not consistently, with adolescents from the middle categories at times scoring higher than high SES adolescents or lower than low SES adolescents. Also, differences in social competences or behavioral control were not observed consistently across all SES indicators. Contrary to our expectations, no differences in social competence and behavioral control were observed between adolescents from different SES variable categories in YOUth (Table 5), l-CID (Table 8, see Supplementary Material), and RADAR (Table 9, see Supplementary Material). Hence, the observed differences in social competence and behavioral control between some SES variable categories in some cohorts fulfilled a second important prerequisite for performing the raking procedure.

Adolescents that are in the lower category on a particular SES indicator (e.g., mother’s education) are not necessarily also in the lower category on another SES indicator (e.g., income). Across all cohorts, the proportion adolescents that is considered to have a low SES background drops sharply if low SES is redefined from being in the lowest category for at least one SES indicator (e.g., having a lower educated mother *or* lower educated father) to being in the lowest category for all SES indicators (e.g., having a lower educated mother *and* lower educated father; see Table 6). Census statistics of the Dutch population have been obtained from different groups of citizens per SES indicator, and are therefore not combined in Table 6.

**Table 6:** Proportions low SES in each cohort differs between definitions.

Cohort	Partial low SES a	Full low SES b
GenR	7.9 %	<1%
L-CID	10.1 %	<1%
NTR	25.9 %	13.1 %
RADAR	35.8 %	12.7 %
TRAILS	53.5 %	4.2 %
YOUth	10.3 %	<1%

a Having a lower educated mother *and/or* father (*and/or* low income).

b Having a lower educated mother *and* father (*and* low income).

### 3.2 Weighted versus unweighted estimates

Our raking procedure yielded no considerable changes, with effect sizes ranging from -0.12 to 0.03 (Table 7). In other words, mean estimates of social competence and behavioral control were mostly similar between the unweighted sample and the weighted counterpart. Similarly, the correlation between social competence and behavioral control was mostly equal in the unweighted sample and weighted sample, with effect sizes ranging from -0.06 to 0.05 (Table 7). Due to a lack of initial differences in adolescent social competence and behavioral control between SES strata, the raking procedure yielded no change in mean scores or correlations in l-CID, RADAR, and YOUth. Despite adolescents from the lower SES categories scoring lower on social competence or behavioral control than adolescents form the higher SES categories, our raking procedure also yielded no changes in normative estimates in Generation R, NTR, and TRAILS.

**Table 7:** Mean item scores (SD), Pearson’s correlations [95 % CI], and effect sizes of social competence and behavioral control in unweighted versus weighted sample.

	Measure		Unweighted					Weighted			Effect size
Social competence *Mean item scores (SD)*											
GenR	CBCL-SP			1.85 (0.19)				1.83 (0.20)			−0.12
L-CID	SDQ-PB			1.68 (0.27)				1.68 (0.26)			0.03
NTR	CBCL-SP			1.83 (0.20)				1.83 (0.20)			0.00
RADAR	R-PB			5.56 (0.89)				5.53 (0.90)			−0.03
TRAILS	CBCL-SP			1.84 (0.21)				1.83 (0.22)			−0.04
YOUth	SDQ-PB			1.70 (0.35)				1.67 (0.40)			−0.10
Behavioral control *Mean item scores (SD)*											
GenR	ASCS			1.65 (0.35)				1.65 (0.36)			0.02
L-CID	BAS-D			1.58 (0.62)				1.55 (0.61)			−0.04
NTR	ASCS			1.62 (0.36)				1.61 (0.35)			−0.01
RADAR	BAS-D			0.87 (0.49)				0.87 (0.50)			0.00
TRAILS	ASCS			1.26 (0.59)				1.25 (0.59)			−0.01
YOUth	ASCS			1.50 (0.39)				1.46 (0.39)			−0.11
Correlation *Social competence, Behavioral control*											
GenR	CBCL-SP, ASCS				0.59		[0.57, 0.61]***		0.64	0.05	
L-CID	SDQ-PB, BAS-D				0.11		[-0.07, 0.28]		0.05	−0.06	
NTR	CBCL-SP, ASCS				0.63		[0.62, 0.65]**		0.63	0.00	
RADAR	R-PB, BAS-D				−0.19		[-0.10, -0.28]**		−0.18	0.01	
TRAILS	CBCL-SP, ASCS				0.55		[0.51, 0.58]**		0.56	0.01	
YOUth	SDQ-PB, ASCS				0.27		[0.18, 0.35]***		0.25	−0.02	

*p < .05.

** *p* < 0.01.

*** *p* < 0.001.

### 3.3 Sensitivity analyses

Sensitivity analyses showed minor but non-significant deviations from the original results after removing outliers (Appendix E). Hence, our raking results are not driven by extreme scores on social competence or behavioral control.

In addition to our preregistered sensitivity analyses, we assessed per cohort whether adolescents that were excluded from analyses due to missing data differed from the included adolescents in our final datasets (Appendix F). In most cohorts, excluded adolescents were from the lower categories on at least one SES indicator, but while in some cohorts the excluded adolescents scored lower on social competence and behavioral control (e.g., TRAILS), in other cohorts scores on social competence and behavioral control were similar between excluded and included adolescents (e.g., RADAR). It must be noted that excluded adolescents per definition had missing data, hence, comparisons to included adolescents are based on a subset of excluded adolescents (i.e., those with available data on the variable of interest).

Furthermore, we reran our raking procedure using all possible combinations of SES variables for weighing (e.g., only using mother’s education; using father’s education and income, etc.) and compared outcomes to our original raking analysis which included all available SES variables. Changes in effect sizes were negligible, suggesting that differences in number of observed SES variables between cohorts does not affect results.

## 4 Discussion

Despite the fact that in 5 of 6 Dutch cohorts participants from lower SES were severely underrepresented and in 3 of the 6 cohorts differences in social competence and behavioral control were observed between SES variable categories, weighting the scores for SES did not produce different normative estimates. In one cohort (i.e., NTR), lower and higher SES adolescents were relatively well-represented, and while differing in social competence and behavioral control, the small sample weights that were applied yielded no difference in normative estimates. Contrary to our expectations, these findings suggest that normative adolescent social competence and behavioral control is not overestimated as a result of predominantly high SES adolescents in Dutch developmental research cohorts.

By testing our research question in 6 different developmental cohorts; with various measures of SES, social competence and behavioral control; on various test statistics; across a broad age range of late childhood and adolescence; and verified through several sensitivity analyses, our findings can be considered robust. However, a number of factors need to be taken into account.

First, it can be questioned how representative the low SES participants in the cohorts are for the low SES population. In the YOUth cohort, for example, only one family was analyzed that is considered ‘low SES’ on all categories (i.e., lower educated mother, lower educated father, and lowest income category). Of the other YOUth families where one of the parents is lower educated, 38.3 % are in the top income category. A similar trend seems to exist in the other cohorts. This suggests that the macro-level sampling bias (i.e., underrepresentation of low SES and overrepresentation of high SES participants) reoccurs on a micro-level (i.e., underrepresentation of lower low SES and overrepresentation of higher low SES participants). When applying a stricter, perhaps more valid definition of ‘low SES’ – requiring being low SES on all SES indicators – some of the cohorts studied here might suffer from underrepresentation and undersampling more than our initial estimates suggest (Table 6). More important, a combination of multiple low SES factors – contrary to the presence of only one low SES factor – has previously been found to hamper psychosocial development (Evans et al., 2013). A considerable proportion of ‘low SES’ adolescents in the 6 cohorts have a single low SES risk factor for development (e.g., low educated mother) which might be compensated or outweighed by high SES protective factors (e.g., high educated father and high income). Hence, it should be questioned whether adolescents that are considered low SES in some cohorts are actually exposed to the developmental adversities that are typically associated with socioeconomic deprivation.

Second, the number of weighing variables was lower than planned. Besides mother’s educational attainment, father’s educational attainment, and family income, we intended to include mother’s occupational level, father’s occupational level, and neighborhood SES as weighing variables for raking. However, parental occupational level is measured differently across cohorts and national census (e.g., classification systems; number of categories; open vs. closed answers), and is currently incompatible. Neighborhood SES was a measure provided by The Netherlands Institute for Social Research (SCP) that got retracted halfway through our study (by SCP itself) due to validity concerns. Especially in the cohorts in which only mother’s and father’s educational attainment has been measured, our operationalization of SES leaves room for improvement. On the contrary, the raking procedure requires observed scores on all weighing variables, hence utilizing fewer SES variables would reduce the number of forced exclusions due to missing data. Furthermore, parental educational attainment is considered a strong indicator of family SES, and driving factor behind other SES indicators such as income and occupation (Erola et al., 2016). Indeed, Dutch census statistics indicate a strong positive association between educational attainment and income (CBS, 2011). However, it must be stressed here that in cohorts that measured both parental educational attainment and family income as SES indicators, a considerable number of adolescents from lower educated parents are still in the higher income categories. The association between educational attainment and income might therefore be different (i.e., weaker) in the cohorts compared to the population, suggesting an atypical low SES participant sample.

Representativeness is not a prerequisite for every research question; some researchers legitimately prioritize balanced sampling over representativeness for testing theories and models of development (Nohr et al., 2013; Rothman et al., 2013). However, compared to smaller, individual studies, large population-based cohort studies may have the additional goal of extrapolating descriptive measures in the sample to the target population. To avoid both undersampling as well as underrepresentation, a sufficient number of lower SES participants in the sample is essential, especially when also considering attrition rates of low SES participants over time (i.e., measurement waves).

For studies on adolescent psychosocial development, we recommend to assess the socioeconomic validity of research samples: 1) by counting the number of SES indicators that are measured, 2) by checking for undersampling in any combination of SES indicators (e.g., lower educated parents and low income; also see Tables 6, and 3) by contrasting the SES characteristics of excluded participants to those of included participants. Measuring SES through multiple indicators (e.g., mother’s education, father’s education, income) instead of a single indicator (e.g., mother’s education) is more accurate in determining which range of the socioeconomic spectrum is – or is not – represented in the research sample (Thaning and Hällsten, 2020). While single SES indicators may have comparable proportions of participants per variable level (e.g., 33 % lower educated mothers; 33 % middle-educated mothers; 33 % higher educated mothers), certain combinations of SES variable levels may still indicate underrepresentation of a socioeconomic group (e.g., 10 % lower educated mothers with low family income; see Table 6). Furthermore, identifying differences between excluded participants and included participants in socioeconomic status as well as in outcomes of interest (see for example Appendix F) is critical to the integrity of research conclusions and to reflect on recruitment and retention quality.

## 5 Conclusion

Estimates of normative social competence and behavioral control in adolescents remained unaffected after raking in 6 Dutch developmental cohorts, despite considerable deviations from the population in SES representativeness and small but significant differences in social competence and behavioral control between some SES strata in some cohorts. These findings are in line with earlier null results between high SES cohorts and population (e.g., Pizzi et al., 2012) and differ from studies that detect an overestimation in developmental outcomes after weighing (e.g., LeWinn et al., 2017). However, our raking procedure was severely limited by the small number of adolescents from the lower categories on all SES variables, high numbers of exclusions due to missing data, and the presumption of having assessed atypically developing low SES adolescents. The question of whether normative estimates of adolescent social competence and behavioral control is overestimated in the Dutch population is therefore not fully answered yet. Replication of our analyses in cohorts with a sufficient number of low SES adolescents, and sufficient variation in combinations of SES variable categories (e.g., equal number of adolescents with lower educated parents from low income families versus adolescents with lower educated parents from high income families) might reveal different estimates of normative social competence and behavioral control after raking. Adolescents with the lowest SES – whose development may in fact be hampered by a network of socioeconomic risk factors and who may potentially benefit most from research findings – largely remain outsiders to developmental research cohorts.

## 6 Preregistration

At the Open Science Framework (OSF), we preregistered the hypotheses and analyses for this study (osf.io/6kzys). The Appendices (A to F) and other supplementary materials, such as Table 8 (L-CID), Table 9 (RADAR), and Table 10 (TRAILS), are also made available here (osf.io/6jtgh). A few deviations from the preregistration must be acknowledged. Given the differences in sample size between cohorts, we redefined outliers on social competence or beha vioral control as scores ≥ (±) 2.58 SD’s from the mean, instead of 3 SD’s. Contrary to our preregistration, no corrections for multiple testing were applied, since a direct comparison of standardized scores was preferred over multiple significance tests. Significance testing between SES strata on social competence and behavioral control (Table 2, Table 3, Table 4, Table 5, Table 6, Table 7) was not preregistered nor corrected for multiple testing given the exploratory nature of the comparisons. These deviations from the preregistration are pragmatic in nature and are expected to have no considerable impact on the outcomes and conclusions of this study.

## Declaration of Competing Interest

None declared.
