# Supplementary material for: Testing sampling bias in estimates of adolescent social competence and behavioral control
Source: Dev Cogn Neurosci. 2020 Oct 22;46:100872. doi: 10.1016/j.dcn.2020.100872 (PMC7642800; doi:10.1016/j.dcn.2020.100872)
Supplement: Supplementary file 1 [file mmc1.docx]

Appendices:

Testing sampling bias in estimates of adolescent social competence and behavioral control

M. Fakkel^1^, M. Peeters^1^, P. Lugtig^1^, M.A.J. Zondervan-Zwijnenburg^1^, E. Blok^2^, T. White^2^, M. van der Meulen^3^, S.T. Kevenaar^4^, G. Willemsen^4^, M. Bartels^4^, D.I. Boomsma^4^, H. Schmengler^2,5^, S. Branje^1^, W.A.M. Vollebergh^1^

^1^Utrecht University, Utrecht, The Netherlands

^2^Erasmus Universiteit, Rotterdam, The Netherlands

^3^Leiden University, The Netherlands

^4^Vrije Universiteit Amsterdam, Amsterdam, The Netherlands

^5^University of Groningen, University Medical Center Groningen, Groningen, The Netherlands

Correspondence to [m.fakkel@uu.nl](mailto:m.fakkel@uu.nl)

**[Index](#Index)**

1. [Categorization of Parental Educational Attainment..…………………………….](#Parental_Education) p. 3
2. [Categorization of Income………………………………………………………...](#Income) p. 7
3. [Correlations between SES indicators in all 6 cohorts……………………………. p. 16](#correlations)
4. [Census statistics per cohort…………………………………………………….....](#Census_statistics) p. 17
5. [Unweighted versus weighted estimates without outliers……....…………………](#without_outliers) p. 18
6. [Differences between excluded vs. included adolescents (per cohort)……………. p. 19](#Excluded_vs_Included)

[click hyperlinks to bookmarks]

A. Categorization of Parental Educational Attainment

Table I.

*Categorization of parental levels of educational attainment into lower, middle, and higher education per cohort and national census.*

|  | **1 – Lower education** | **2 – Middle education** | **3 – Higher education** |
| --- | --- | --- | --- |
| **National census** | 1 = Primary education  2 = Vmbo-b/k, mbo1  3 = Vmbo-g/t, havo-, vwo-onderbouw | 4 = Havo, vwo  5 = Mbo2 en mbo3  6 = Mbo4 | 7 = Hbo-, wo-bachelor  8 = Hbo-, wo-master, doctor |
| **GenR** | 1 = No primary school completed  2 = Primary school, Special primary school, Special secondary school  3 = Pre-vocational and lower secondary education | 4 = General secondary education (HAVO/VWO)  5 = Senior secondary vocational education (MBO) | 6 = Higher professional (HBO); University |
| **L-CID** | 1 = Elementary school/Primary education  2 = VMBO, MAVO, LBO, LTS, VSO, or equivalent | 3 = HAVO, VWO, Gymnasium, MBO, MTS, MEAO, or equivalent | 4 = HBO, HTS, propedeuse or bachelor University education  5 = Master University education, post-HBO education |
| **NTR** | 1 = Elementary school  2 = Several years of lower general secondary school (mulo, mavo)  3 = Graduated from lower general secondary school  4 = Several years of lower vocational training (lts, domestic science school)  5 = Graduated from lower vocational training (lts, domestic science school)  6 = Several years of upper general secondary school (havo, vwo or hbs, athenaeum)  7 = Graduated from upper general secondary school (havo, vwo or hbs, athenaeum)  8 = Several years of intermediate vocational education | 9 = Intermediate vocational education completed  10 = Several years of higher vocational education or university (hbo) | 11 = Higher vocational education completed (hbo)  12 = University degree  13 = Post-graduate degree or PhD |
| **RADAR** | 1 = None  2 = Kindergarten  3 = Primary education  4 = Special primary education (BLO, LOM)  5 = Special primary education (ZMOK, ZLK, ZMLK)  6 = School for physical, visual, or auditive impaired  7 = Higher special education (VSO-LOM, VSO-MLK)  8 = Lower secondary education (VBO)  9 = Lower secondary education (VMBO)  10 = Lower secondary education (MULO, MAVO, LAVO) | 11=Higher secondary education (HAVO, VWO, HBS, MMS)  12 = Tertiary vocational education (MBO) | 13 = Higher vocational education  14 = University or post-HBO education |
| **TRAILS** | 1= No education  2= Primary (special) education  3= Lower secondary vocational education or equivalent (vglo, lavo, lbo, lts, lhno, huishoudschool, leao, ulo, mulo/mavo, at least 3 years havo/vwo (but not graduated), secondary special education) | 4= Higher secondary education (hbs, mms, gymnasium, havo, vwo, mbo, mts, meao, leerlingwezen) | 5= Higher education first degree (hbo, propedeuse or bachelor university education)  6= Higher education second degree (Master onderwijs, enginering degree, post-hbo education)  7= Hoger onderwijs derde trap (tweede fase opleiding, post-doctorale opleiding, promotie) |
| **YOUth** | 1 = Primary education (BAO)  2 = Special primary education (SBAO)  3 = (Secondary) Special education ((V)SO)  4 = Practical education (PRO)  5 = Secondary vocational education ‘basis/kader’ (VMBO-BK)  6 = Secondary vocational education ‘theoretische leergang’ (VMBO-TL)  7 = Higher general secondary education (HAVO)  8= Higher secondary education (VWO) | 9 = Tertiary vocational education (MBO) | 10 = Higher vocational education (HBO)  11= University education (WO) |

*
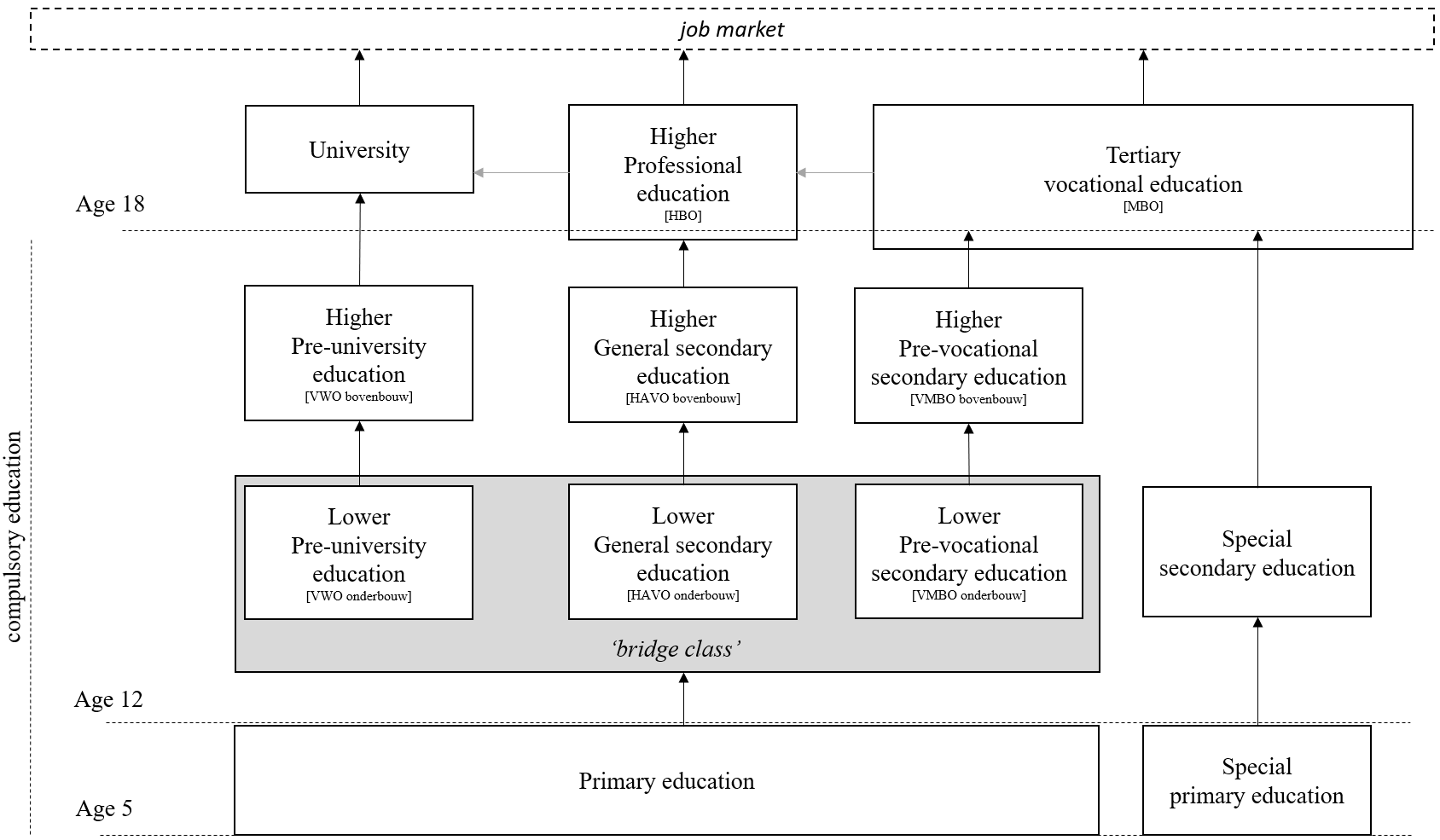
*

Figure 1. *Simplified diagram of Dutch educational system corresponding to our educational classification approach.*

[**[back to index]**](#Index)

B. Categorization of Income

Income of the Dutch population was measured in the census as mean (gross or net) annual income of deciles (= equally sized 10% groups of households), corresponding to the year of data collection in the cohort. To create matching categories, we looked at census and cohort boundaries that fall closest to each other, and result in 5 categories with preferably more than 10% and less than 50% of the population (cohort proportions were fixed, though categories could be collapsed). None of the cohort income boundaries exactly matched census income boundaries, but given the mean income of the 10% groups in the population, we estimated the population percentage that is expected to be in each cohort category. According to the census statistics, the decile mean incomes are also the decile median incomes (i.e., normally distributed deciles). This is true for all deciles, expect for the lowest decile (i.e., median income higher than mean income) and highest decile (i.e., median income lower than mean income). The other 8 deciles could reliably be split into percentiles if necessary, to match census income boundaries to cohort income boundaries.

**Generation R**:

Table II.

Original answer categories of net income in Generation R and percentage of adolescents (*n* = 3898) per category.

| **Category** | **Income** | **%** |
| --- | --- | --- |
|  | < €1.200 | 4.0% |
|  | €1.200 – 2.000 | 10.4% |
|  | €2.000 – 3.200 | 14.9% |
|  | €3.200 – 4.000 | 19.4% |
|  | > €4.000 | 51.4% |

Table III.

Mean net income per population decile of the Netherlands (2012).

| **Deciles** | **Income** | **Deciles** | **Income** |
| --- | --- | --- | --- |
| 1e 10% | € 569 | 6e 10% | € 2623 |
| 2e 10% | € 1231 | 7e 10% | € 3077 |
| 3e 10% | € 1554 | 8e 10% | € 3600 |
| 4e 10% | € 1869 | 9e 10% | € 4354 |
| 5e 10% | € 2223 | 10e 10% | € 7185 |

Figure 2.

*Mean net income per population decile of the Netherlands (2012).*


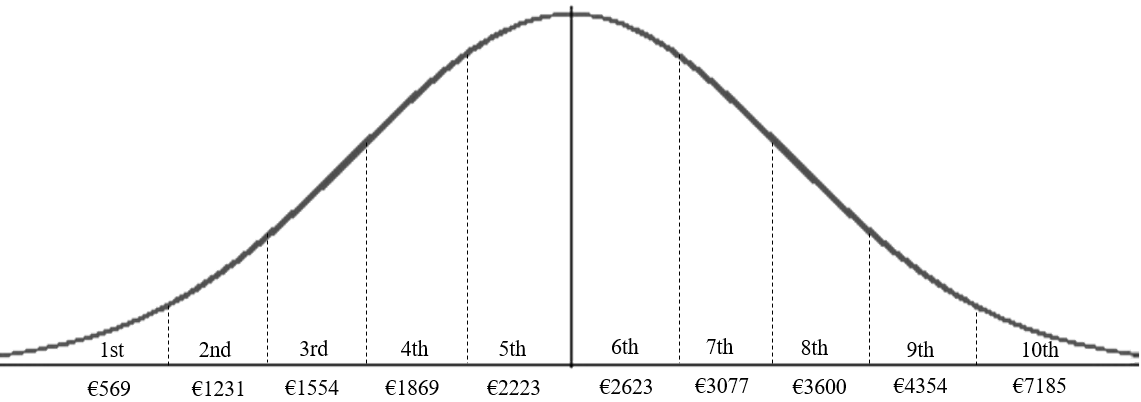


Next, the census data was matched to these cohort income categories to obtain population percentages (rounded off to 0.5).

1. The lowest cohort income boundary of €1200 falls between the mean incomes of the 1^st^ and 2^nd^ population deciles. If the mean income of the 1^st^ decile is €569 and the mean income of the 2^nd^ decile is €1231, and income is normally distributed in these two deciles, then the mean income is also the median income, meaning that €569 is also the mean income of the 5^th^ percentile and €1231 the mean income of the 15^th^ percentile: €1231 minus €569 = €662 divided by 10 (15 minus 5) = €66,20 increase per percentile.

€1231 minus €1200 = €31 / €66,20 ≈ 0.5% 🡪 15% - 0.5% = 14.5%

€1200 minus €569 = €631 / €66,20 ≈ 9.5%. 🡪 5% + 9.5% = 14.5%

So, 14.5% of the population has an income of < €1200.

1. The second cohort income boundary of €2000 falls between mean incomes of the 4^th^ and 5^th^ population deciles: €1869is the mean income of the 35^th^ percentile and €2223 is the mean income of the 45^th^ percentile.

€2223 minus €1869= €354 / 10 = €35,40 per percentile.

€2223 minus €2000 = €223 / €35,40 ≈ 6.3% 🡪 45% - 6.3% ≈ 38%

€2000 minus €1869= €131 / €35,40 ≈ 3.7% 🡪 35% + 3.7% ≈ 38%

So, 38.7% minus 14.5% = 24.2% of the population has an income of €1200-€2000.

1. The third cohort income boundary of €3200 falls between mean incomes of the 7^th^ and 8^th^ population deciles: €3077is the mean income of the 65^th^ percentile and €3600 is the mean income of the 75^th^ percentile.

€3600 minus €3077= €523 / 10 = €52,30 per percentile.

€3600 minus €3200 = €400 / €52,30 ≈ 7.7% 🡪 75% - 7.7% ≈ 67%

€3200 minus €3077= €123 / €52,30 ≈ 2.3% 🡪 65% + 2.3% ≈ 67%

So, 67% minus 38% = 29.0% of the population has an income of €2000-€3200.

1. The fourth cohort income boundary of €4000 falls between mean incomes of the 8^th^ and 9^th^ population decile: €3600 is the mean income of the 75^th^ percentile and €4354 is the mean income of the 85^th^ percentile.

€4354 minus €3600 = €754 / 10 = €74,50 per percentile.

€4354 minus €4000 = €354 / €74,50 ≈ 4.8% 🡪 85% - 4.8% ≈ 80%

€4000 minus €3600 = €400 / €74,50 ≈ 5.4% 🡪 75% + 5.1% ≈ 80%

So, 80% minus 67% = 13.0% of the population has an income of €3200-€4000.

1. Given the above calculations, 19.5% of the population is in the top income category of > €4000.

Figure 3.

*Matching population boundaries to cohort boundaries of income. Percentages of Dutch population per category.*


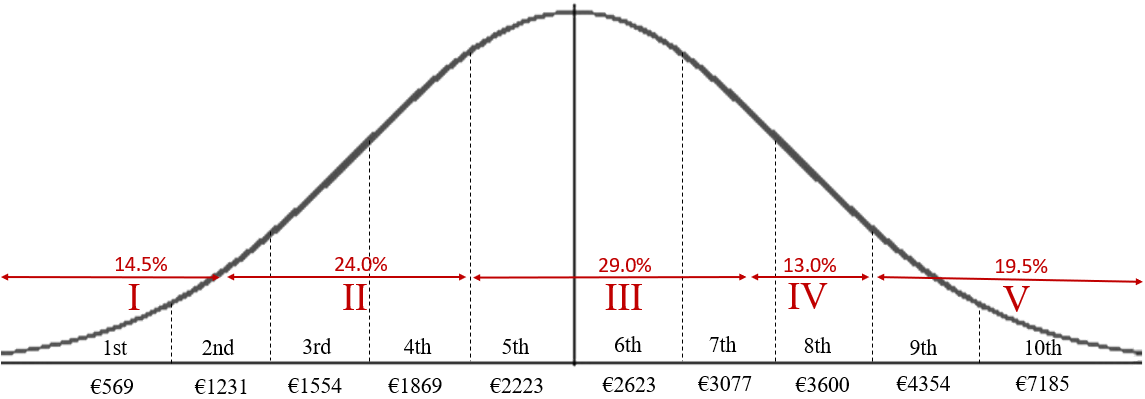
Table IV.

Percentage of Generation R adolescents and Dutch population per income category.

| **Category** | **Income** | **% GenR** | **% Dutch population** |
| --- | --- | --- | --- |
|  | <€1135 | 4.0% | 14.5% |
|  | €1135 – €1590 | 10.4% | 24.0% |
|  | €1590 – €2045 | 14.9% | 29.0% |
|  | €2045 – €2955 | 19.4% | 13.0% |
|  | >€2955 | 51.4% | 19.5% |

**TRAILS**:

Table V.

Original answer categories of net income in TRAILS and percentage of adolescents (*n* = 1535) per category.

| **Category** | **Income** | **%** |
| --- | --- | --- |
| 1 | <€680 | 0.9% |
| 2 | €680 – €1135 | 15.9% |
| 3 | €1135 – €1590 | 18.1% |
| 4 | €1590 – €2045 | 21.4% |
| 5 | €2045 – €2500 | 17.4% |
| 6 | €2500 – €2955 | 11.9% |
| 7 | €2955 – €3410 | 6.4% |
| 8 | €3410 – €3865 | 4.5% |
| 9 | >€3865 | 3.6% |

Given the above-mentioned considerations for creating matching categories, the original cohort categories were clustered as:

Table VI.

Clustered income categories TRAILS.

| **Category** | **Income** | **%** |
| --- | --- | --- |
|  | < €1135 | 7.0% |
|  | €1135 – €1590 | 16.9% |
|  | €1590 – €2045 | 23.7% |
|  | €2045 – €2955 | 35.1% |
|  | > €2955 | 17.3% |

Table VII.

Mean net income per population decile of the Netherlands (2001).

| **Deciles** | **Income** | **Deciles** | **Income** |
| --- | --- | --- | --- |
| 1e 10% | € 500 | 6e 10% | € 1985 |
| 2e 10% | € 970 | 7e 10% | € 2300 |
| 3e 10% | € 1200 | 8e 10% | € 2675 |
| 4e 10% | € 1550 | 9e 10% | € 3225 |
| 5e 10% | € 1690 | 10e 10% | € 5185 |

Figure 4.

*Mean net income per population decile of the Netherlands (2001).*


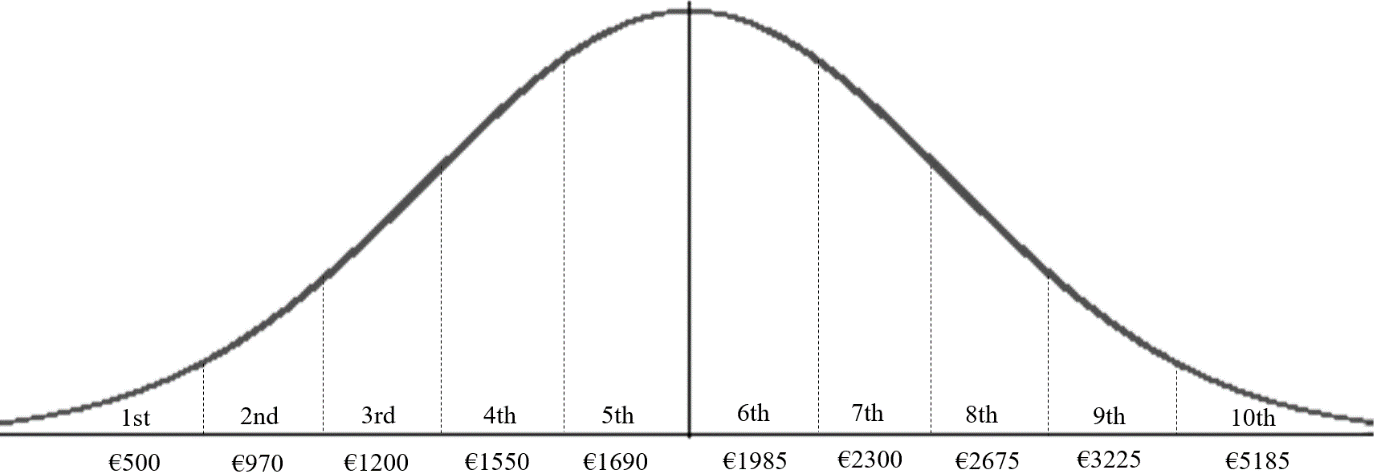


Next, the census data was matched to these cohort income categories to obtain population percentages.

1. The lowest cohort income boundary of €1135 falls between the mean incomes of the 2^nd^ and 3^rd^ population deciles. If the mean income of the 2^nd^ decile is €970 and the mean income of the 3^rd^ decile is €1200, and income is normally distributed in these two deciles, then the mean income is also the median income, meaning that €970 is also the mean income of the 15^th^ percentile and €1200 the mean income of the 25^th^ percentile: €1200 minus €970 = €230 divided by 10 (25 minus 15) = €23 increase per percentile.

€1200 minus €1135 = €65 / €23 ≈ 2.8% 🡪 25% - 2.8% = 22.2%

€1135 minus €970 = €165 / €23 ≈ 7.2%. 🡪 15% + 7.2% = 22.2%

So, 22.2% of the population has an income of < €1135.

1. The second cohort income boundary of €1590 falls between mean incomes of the 4^th^ and 5^th^ population deciles: €1550 is the mean income of the 35^th^ percentile and €1690 is the mean income of the 45^th^ percentile.

€1690 minus €1550 = €140 / 10 = €14 per percentile.

€1690 minus €1590 = €100 / €14 ≈ 7.1% 🡪 45% - 7.1% = 37.9%

€1590 minus €1550 = €40 / €14 ≈ 2.9% 🡪 35% + 2.9% = 37.9%

So, 37.9% minus 22.2% = 15.7% of the population has an income of €1135-€1590.

1. The third cohort income boundary of €2045 falls between mean incomes of the 6^th^ and 7^th^ population deciles: €1985 is the mean income of the 55^th^ percentile and €2300 is the mean income of the 65^th^ percentile.

€2300 minus €1985 = €315 / 10 = €31,50 per percentile.

€2300 minus €2045 = €255 / €31,50 ≈ 8.1% 🡪 65% - 8.1% = 56.9%

€2045 minus €1985 = €60 / €31,50 ≈ 1.9% 🡪 55% + 1.9% = 56.9%

So, 56.9% minus 37.9% = 19.0% of the population has an income of €1590-€2045.

1. The fourth cohort income boundary of €2955 falls between mean incomes of the 8^th^ and 9^th^ population decile: €2675 is the mean income of the 75^th^ percentile and €3225 is the mean income of the 85^th^ percentile.

€3225 minus €2675 = €550 / 10 = €55 per percentile.

€3225 minus €2955 = €270 / €55 ≈ 4.9% 🡪 85% - 4.9% = 80.1%

€2955 minus €2675 = €280 / €55 ≈ 5.1% 🡪 75% + 5.1% = 80.1%

So, 80.1% minus 56.9% = 23.2% of the population has an income of €2045-€2955.

1. Given the above calculations, 19.9% of the population is in the top income category of > €2955.

Figure 5.

*
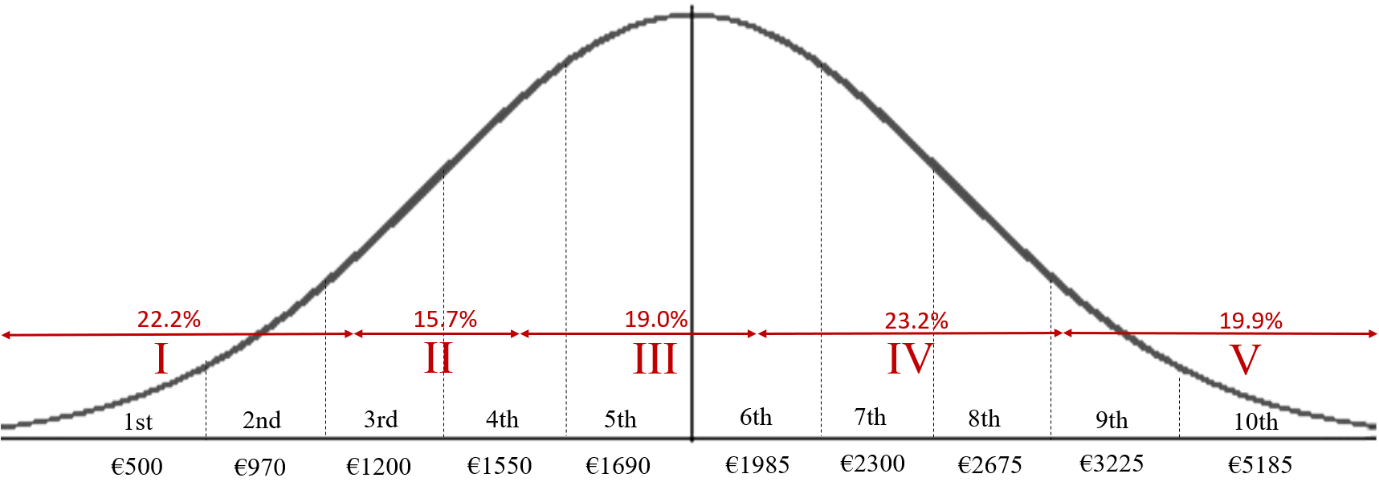
Matching population boundaries to cohort boundaries of income. Percentages of Dutch population per category.*

Table VIII.

*Percentage of TRAILS adolescents and Dutch population per income category.*

| **Category** | **Income** | **% TRAILS** | **% Dutch population** |
| --- | --- | --- | --- |
|  | <€1135 | 7.0% | 22.2% |
|  | €1135 – €1590 | 16.9% | 15.7% |
|  | €1590 – €2045 | 23.7% | 19.0% |
|  | €2045 – €2955 | 35.1% | 23.2% |
|  | >€2955 | 17.3% | 19.9% |

**YOUth**:

Table IX.

*Original answer categories of gross income in YOUth and percentage of adolescents (n = 595) per category.*

| **Category** | **Income** | **%** |
| --- | --- | --- |
| 1 | <€1250 | 1.5% |
| 2 | €1250 – €2000 | 4.0% |
| 3 | €2000 – €3000 | 6.4% |
| 4 | €3000 – €4000 | 15.8% |
| 5 | >€4000 | 72.3% |

Table X.

*Median gross income per population decile of the Netherlands (2015).*

| **Deciles** | **Income** | **Deciles** | **Income** |
| --- | --- | --- | --- |
| 1e 10% | € 1008 | 6e 10% | € 4215 |
| 2e 10% | € 1610 | 7e 10% | € 5185 |
| 3e 10% | € 2110 | 8e 10% | € 6370 |
| 4e 10% | € 2685 | 9e 10% | € 8040 |
| 5e 10% | € 3395 | 10e 10% | € 11560 |

Figure 6.

*
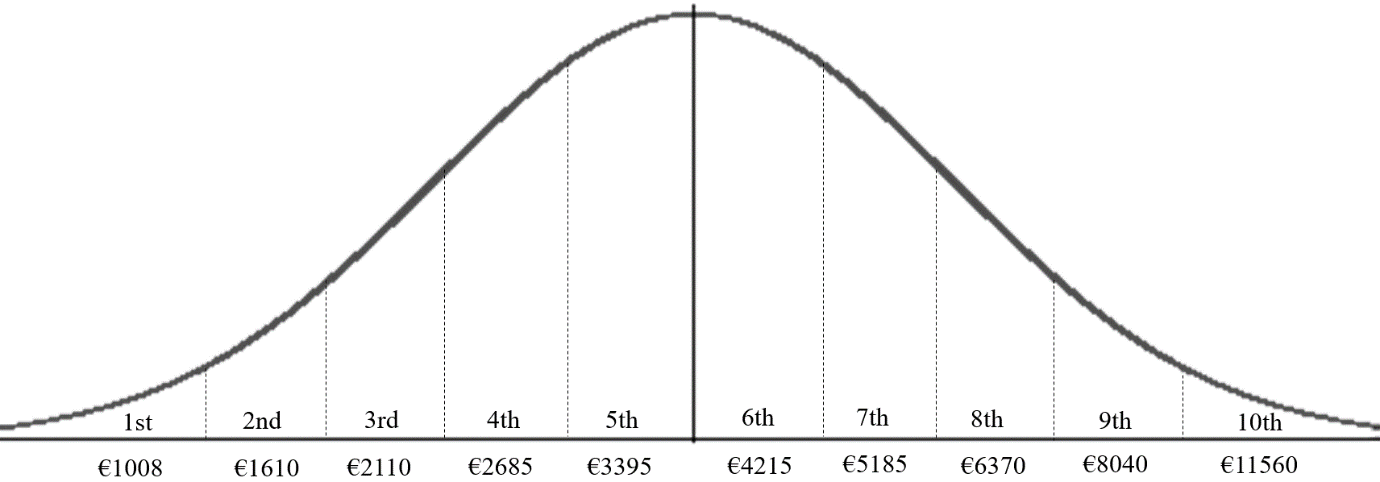
Median gross income per population decile of the Netherlands (2015).*

Next, the census data was matched to these cohort income categories to obtain population percentages.

1. The lowest cohort income boundary of €1250 falls between the median incomes of the 1^st^ and 2^nd^ population deciles: €1008 is also the mean income of the 5^th^ percentile and €1610 the mean income of the 15^th^ percentile: €1610 minus €1008 = €602 divided by 10 (25 minus 15) = €60,20 increase per percentile.

€1610 minus €1250 = €360 / €60,20 ≈ 6.0% 🡪 15% - 6.0% = 9.0%

€1250 minus €1008 = €242 / €60,20 ≈ 4.0%. 🡪 5% + 4.0% = 9.0%

So, 9.0% of the population has an income of < €1250.

1. The second cohort income boundary of €2000 falls between mean incomes of the 2^nd^ and 3^rd^ population deciles: €1610 is the mean income of the 15^th^ percentile and €2110 is the mean income of the 25^th^ percentile.

€2110 minus €1610 = €500 / 10 = €50 per percentile.

€2110 minus €2000 = €110 / €50 ≈ 2.2% 🡪 25% - 2.2% = 22.8%

€2000 minus €1610 = €390 / €50 ≈ 7.8% 🡪 15% + 7.8% = 22.8%

So, 22.8% minus 9.0% = 13.8% of the population has an income of €1250-€2000.

1. The third cohort income boundary of €3000 falls between mean incomes of the 4^th^ and 5^th^ population deciles: €2685 is the mean income of the 35^th^ percentile and €3395 is the mean income of the 45^th^ percentile.

€3395 minus €2685 = €710 / 10 = €71 per percentile.

€3395 minus €3000 = €395 / €71 ≈ 5.6% 🡪 45% - 5.6% = 39.4%

€3000 minus €2685 = €315 / €71 ≈ 4.4% 🡪 35% + 4.4% = 39.4%

So, 39.4% minus 22.8% ≈ 16.6% of the population has an income of €2000-€3000.

1. The fourth cohort income boundary of €4000 falls between mean incomes of the 5^th^ and 6^th^ population decile: €3395 is the mean income of the 45^th^ percentile and €4215 is the mean income of the 55^th^ percentile.

€4215 minus €3395 = €820 / 10 = €82 per percentile.

€4215 minus €4000 = €215 / €82 ≈ 2.6% 🡪 55% - 2.6% = 52.4%

€4000 minus €3395 = €605 / €82 ≈ 7.4% 🡪 45% + 7.4% = 52.4%

So, 52.4% minus 39.4% = 13.0% of the population has an income of €3000-€4000.

1. Given the above calculations, 47.6% of the population is in the top income category of > €4000.

Figure 7.

*Matching population boundaries to cohort boundaries of income. Percentages of Dutch population per category.*


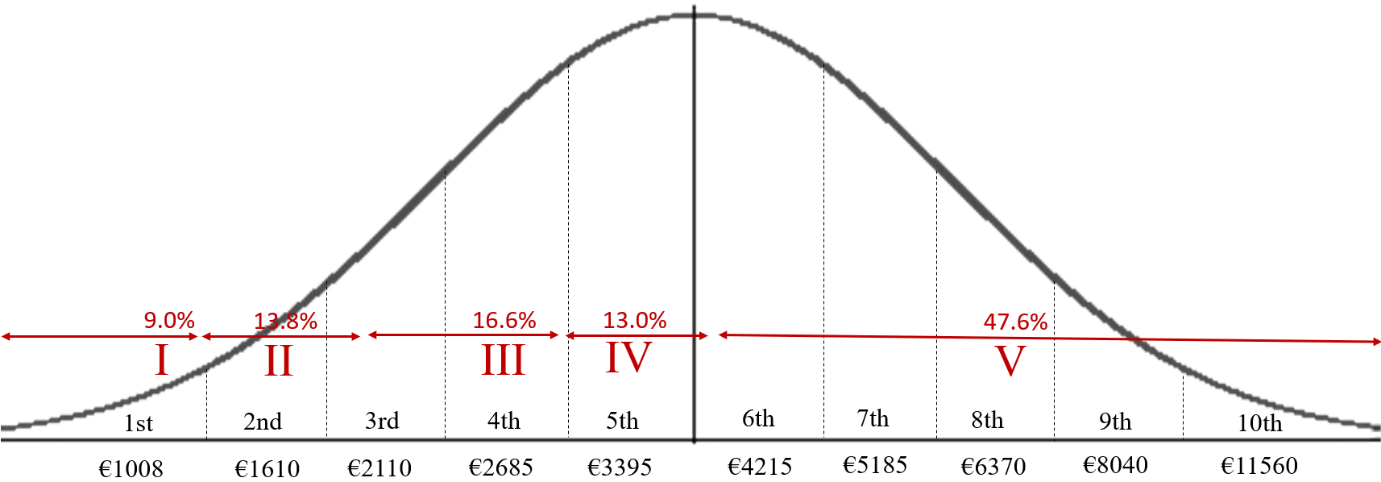


Table XI.

*Percentage of YOUth adolescents and Dutch population per income category.*

| **Category** | **Income** | **% YOUth** | **% Dutch population** |
| --- | --- | --- | --- |
|  | <€1250 | 1.5% | 9.0% |
|  | €1250 – €2000 | 4.0% | 13.8% |
|  | €2000 – €3000 | 6.4% | 16.6% |
|  | €3000 – €4000 | 15.8% | 13.0% |
|  | >€4000 | 72.3% | 47.6% |

[**[back to index]**](#Index)

C. Correlations between SES indicators in all 6 cohorts

Table XII.

*Correlations between SES indicators in all 6 cohorts.*

***p < .01*

|  | 1 | | 2 | | | | 3 | |
| --- | --- | --- | --- | --- | --- | --- | --- | --- |
| 1. Mother’s education |  | | |  | | |  | |
| 1. Father’s education | GenR  L-CID  NTR  RADAR  TRAILS  YOUth | 0.48**  0.31**  0.46**  0.50**  0.54**  0.35** | |  | |  |  |  |
| 1. Income | GenR  TRAILS  YOUth | 0.39**  0.44**  0.24** | | GenR  TRAILS  YOUth | 0.41**  0.52**  0.29** | |  | |

[**[back to index]**](#Index)

D. Census statistics per cohort

Table XIII.

*URL’s to open source database from Statistics Netherlands. Last checked on January 24^th^, 2020.*

| **Cohort** | **URL’s** |
| --- | --- |
| GenR | [Parental education](https://opendata.cbs.nl/statline/#/CBS/nl/dataset/82275NED/table?dl=29D77)  [Income](https://opendata.cbs.nl/statline/#/CBS/nl/dataset/83932NED/table?dl=273D3) |
| L-CID | [Parental education](https://opendata.cbs.nl/statline/#/CBS/nl/dataset/82275NED/table?dl=273C7) |
| NTR | [Parental education](https://opendata.cbs.nl/statline/#/CBS/nl/dataset/82275NED/table?dl=2F7B8) |
| RADAR | [Parental education](https://opendata.cbs.nl/statline/#/CBS/nl/dataset/82275NED/table?dl=2F81A) |
| TRAILS | [Parental education](https://opendata.cbs.nl/statline/#/CBS/nl/dataset/71013ned/table?dl=2F849)  [Income](https://opendata.cbs.nl/statline/#/CBS/nl/dataset/71013ned/table?dl=304ED) |
| YOUth | [Parental education](https://opendata.cbs.nl/statline/#/CBS/nl/dataset/82275NED/table?dl=28441)  [Income](https://opendata.cbs.nl/statline/#/CBS/nl/dataset/83932NED/table?dl=2B787) |

[**[back to index]**](#Index)

E. Unweighted versus weighted estimates without outliers

Table XIV.

|  | **Measure** | | **Unweighted** | | | | | | **Weighted** | | **Effect size** |
| --- | --- | --- | --- | --- | --- | --- | --- | --- | --- | --- | --- |
| **Social competence**  *Mean item scores (SD)* | |  | | | | |  | |  | |  |
| GenR | CBCL-SP | | | 1.85 (0.19) | | | | | 1.83 (0.20) | | -0.12 |
| L-CID | SDQ-PB | | | 1.68 (0.27) | | | | | 1.68 (0.26) | | 0.03 |
| NTR | CBCL-SP | | | 1.83 (0.20) | | | | | 1.83 (0.20) | | 0.00 |
| RADAR | R-PB | | | 5.56 (0.89) | | | | | 5.53 (0.90) | | -0.03 |
| TRAILS | CBCL-SP | | | 1.84 (0.21) | | | | | 1.83 (0.22) | | -0.04 |
| YOUth | SDQ-PB | | | 1.70 (0.35) | | | | | 1.67 (0.40) | | -0.10 |
| **Behavioral control**  *Mean item scores (SD)* | |  | | | | |  | |  | |  |
| GenR | ASCS | | | 1.65 (0.35) | | | | | 1.65 (0.36) | | 0.02 |
| L-CID | BAS-D | | | 1.58 (0.62) | | | | | 1.55 (0.61) | | -0.04 |
| NTR | ASCS | | | 1.62 (0.36) | | | | | 1.61 (0.35) | | -0.01 |
| RADAR | BAS-D | | | 0.87 (0.49) | | | | | 0.87 (0.50) | | 0.00 |
| TRAILS | ASCS | | | 1.58 (0.38) | | | | | 1.57 (0.36) | | -0.01 |
| YOUth | ASCS | | | 1.50 (0.39) | | | | | 1.45 (0.39) | | -0.14 |
| **Correlation**  *Social competence, Behavioral control* | | | | | |  | | | |  |  |
| GenR | CBCL-SP, ASCS | | | | 0.52 | | | [0.49, 0.54] *** | | 0.57 | 0.05 |
| L-CID | SDQ-PB, BAS-D | | | | 0.10 | | | [-0.08, 0.27] | | 0.06 | -0.04 |
| NTR | CBCL-SP, ASCS | | | | 0.57 | | | [0.56, 0.60] | | 0.58 | 0.00 |
| RADAR | R-PB, BAS-D | | | | -0.23 | | | [-0.32, -0.14]*** | | -0.23 | 0.00 |
| TRAILS | CBCL-SP, ASCS | | | | 0.55 | | | [0.49, 0.57]*** | | 0.53 | 0.00 |
| YOUth | SDQ-PB, ASCS | | | | 0.24 | | | [0.15, 0.32]*** | | 0.19 | -0.05 |

*Mean item scores (SD), correlations [95% CIs], and effect sizes of social competence and behavioral control in unweighted versus weighted sample without outliers.*

* *p* < .05, ** *p* < .01, *** *p* < .001

[**[back to index]**](#Index)

F. Differences between excluded vs. included adolescents (per cohort)

For the raking procedure, it is essential that adolescents have observations on all weighting variables (i.e., SES variables), and on social competence *or* behavioral control.

These inclusion criteria divided each original dataset into two: adolescents with full information that were to be ‘included’ versus adolescents with missing information that were to be ‘excluded’. Per cohort, we checked whether excluded adolescents differed from included adolescents on the available SES indicators and measures of social competence and behavioral control. Note that the excluded adolescents per definition had missing information, and that each comparison with included adolescents is based on a *subset* of excluded adolescents (i.e., those with available data on the variable of interest).

**GenR:**

*How many adolescents had to be excluded?*

2875 of the 6770 adolescents (42.5%) were excluded for the raking procedure due to missing observations.

*Do excluded adolescents differ in* ***mother’s educational attainment*** *from included adolescents?*

2360 of the 2875 excluded adolescents (82.1%) had reported mother’s educational attainment. Excluded adolescents tend to have lower educated mothers than included adolescents.

*Do excluded adolescents differ in* ***father’s educational attainment*** *from included adolescents?*

1799 of the 2875 excluded adolescents (62.6%) had reported father’s educational attainment. Excluded adolescents tend to have lower father’s education than included adolescents.

*Do excluded adolescents differ in* ***income*** *from included adolescents?*

875 of the 2875 excluded adolescents (30.4%) had reported income. These excluded adolescents were from lower income families than included adolescents.

*Do excluded adolescents differ in* ***SocialCompetence_CBCL*** *from included adolescents?*

1053 of the 2875 excluded adolescents (36.6%) had a score on SocialCompetence_CBCL. Excluded adolescents scored lower on SocialCompetence_CBCL than included adolescents.

*Do excluded adolescents differ in* ***BehavioralControl_ASCS*** *from included adolescents?*

1041 of the 2875 excluded adolescents (36.2%) had a score on BehavioralControl_ASCS. These excluded adolescents scored lower on BehavioralControl_ASCS than included adolescents.

**Conclusion**: Excluded GenR adolescents have lower educated mothers and fathers and are from lower income families than included adolescents; and score lower on SocialCompetence_CBCL and BehavioralControl_ASCS.

**L-CID:**

*How many adolescents had to be excluded?*

14 of the 156 adolescents (9.0%) were excluded for the raking procedure due to missing observations.

*Do excluded adolescents differ in* ***mother’s educational attainment*** *from included adolescents?*

All excluded adolescents had reported mother’s educational attainment. Excluded adolescents tend to have lower educated mothers than included adolescents, though too few cases were available to draw strong conclusions.

*Do excluded adolescents differ in* ***father’s educational attainment*** *from included adolescents?*

10 of the 14 excluded adolescents (71.4%) had reported father’s educational attainment. Excluded adolescents tend to be similar in father’s education to included adolescents, though too few cases were available to draw strong conclusions.

*Do excluded adolescents differ in* ***SocialCompetence_SDQ*** *from included adolescents?*

3 of the 14 excluded adolescents (21.4%) had a score on SocialCompetence_SDQ. Excluded adolescents scored similarly on SocialCompetence_SDQ than included adolescents, though too few cases were available to draw strong conclusions.

*Do excluded adolescents differ in* ***BehavioralControl_BASD*** *from included adolescents?*

Only 1 of the 14 excluded adolescents (7.1%) had a score on BehavioralControl_BASD. This excluded adolescent scored similarly on BehavioralControl_BASD as included adolescents, though no strong conclusions can be drawn from one single case.

**Conclusion**: Excluded L-CID adolescents have lower educated mothers but similarly educated fathers as included adolescents; and score similarly on SocialCompetence_SDQ and BehavioralControl_BASD, though note the small number of excluded cases.

**NTR**:

*How many adolescents had to be excluded?*

Of the 15270 adolescents in the original dataset of singletons, twins, or triplets; we randomly selected one adolescent per household = 7635. Of these 7635 adolescents, 1369 (17.9%) were excluded for the raking procedure due to missing observations.

*Do excluded adolescents differ in* ***mother’s educational attainment*** *from included adolescents?*

1176 of the 1369 excluded adolescents (85.9%) had reported mother’s educational attainment. Excluded adolescents tend to have lower educated mothers than included adolescents.

*Do excluded adolescents differ in* ***father’s educational attainment*** *from included adolescents?*

724 of the 1369 excluded adolescents (52.9%) had reported father’s educational attainment. Excluded adolescents tend to have lower educated fathers than included adolescents.

*Do excluded adolescents differ in* ***SocialCompetence_CBCL*** *from included adolescents?*

722 of the 1369 excluded adolescents (52.7%) had a score on SocialCompetence_CBCL. Excluded adolescents scored lower on SocialCompetence_CBCL than included adolescents.

*Do excluded adolescents differ in* ***BehavioralControl_ASCS*** *from included adolescents?*

716 of the 1369 excluded adolescents (52.3%) had a score on BehavioralControl_ASCS. Excluded adolescents scored lower on BehavioralControl_ASCS than included adolescents.

**Conclusion**: Excluded NTR adolescents have lower educated mothers and fathers than included adolescents; and score lower on SocialCompetence_CBCL and BehavioralControl_ASCS.

**RADAR**:

*How many adolescents had to be excluded?*

56 of 497 adolescents (11.3%) were excluded for the raking procedure due to missing observations.

*Do excluded adolescents differ in* ***mother’s educational attainment*** *from included adolescents?*

52 of the 56 excluded adolescents (94.6%) had reported mother’s educational attainment. These excluded adolescents tend to have lower educated mothers than included adolescents.

*Do excluded adolescents differ in* ***father’s educational attainment*** *from included adolescents?*

Only 4 of the 56 excluded adolescents (7.1%) had reported father’s educational attainment. Though father’s educational attainment was similar between these excluded and included adolescents, the number of cases is too small to make meaningful comparisons.

*Do excluded adolescents differ in* ***SocialCompetence_RPB*** *from included adolescents?*

52 of the 56 excluded adolescents (94.6%) had a score on SocialCompetence_RPB. These excluded adolescents scored similarly on SocialCompetence_RPB as included adolescents.

*Do excluded adolescents differ in* ***BehavioralControl_BASD*** *from included adolescents?*

51 of the 56 excluded adolescents (91.1%) had a score on BehavioralControl_BASD. These excluded adolescents scored similarly on BehavioralControl_BASD as included adolescents.

**Conclusion**: Excluded RADAR adolescents have lower educated mothers but similarly educated fathers as included adolescents; and scored similarly on SocialCompetence_RPB and BehavioralControl_BASD.

**TRAILS**:

*How many adolescents had to be excluded?*

694 of 2229 adolescents (31.1%) were excluded for the raking procedure due to missing observations.

*Do excluded adolescents differ in* ***mother’s educational attainment*** *from included adolescents?*

478 of the 694 excluded adolescents (68.9%) had reported mother’s educational attainment. These excluded adolescents tend to have lower educated mothers than included adolescents.

*Do excluded adolescents differ in* ***father’s educational attainment*** *from included adolescents?*

224 of the 694 excluded adolescents (32.3%) had reported father’s educational attainment. These excluded adolescents tend to have similarly educated fathers as included adolescents.

*Do excluded adolescents differ in* ***income*** *from included adolescents?*

472 of the 694 excluded adolescents (68.0%) had reported income. These excluded adolescents tend to be from lower income families than included adolescents.

*Do excluded adolescents differ in* ***SocialCompetence_CBCL*** *from included adolescents?*

479 of the 694 excluded adolescents (69.0%) had a score on SocialCompetence_CBCL. These excluded adolescents scored lower on SocialCompetence_CBCL than included adolescents.

*Do excluded adolescents differ in* ***BehavioralControl_BASD*** *from included adolescents?*

580 of the 694 excluded adolescents (83.6%) had a score on BehavioralControl_BASD. These excluded adolescents scored similarly on BehavioralControl_BASD as included adolescents.

**Conclusion**: Excluded TRAILS adolescents have lower educated mothers, similarly educated fathers, and are from lower income families than included adolescents; while also scoring lower on SocialCompetence_CBCL and on BehavioralControl_ASCS (but not on main measure BehavioralControl_BASD) than included adolescents.

**YOUth**:

*How many adolescents had to be excluded?*

235 of 830 adolescents (28.3%) were excluded for the raking procedure due to missing observations.

*Do excluded adolescents differ in* ***mother’s educational attainment*** *from included adolescents?*

215 of the 235 excluded adolescents (91.5%) had reported mother’s educational attainment. These excluded adolescents had similarly educated mothers as included adolescents.

*Do excluded adolescents differ in* ***father’s educational attainment*** *from included adolescents?*

36 of the 235 excluded adolescents (15.3%) had reported father’s educational attainment. These excluded adolescents had similarly educated fathers as included adolescents.

*Do excluded adolescents differ in* ***income*** *from included adolescents?*

207 of the 235 excluded adolescents (88.1%) had reported income. These excluded adolescents were from lower income families than included adolescents.

*Do excluded adolescents differ in* ***SocialCompetence_SDQ*** *from included adolescents?*

202 of the 235 excluded adolescents (86.0%) had reported father’s educational attainment. These excluded adolescents scored similarly on SocialCompetence_SDQ as included adolescents.

*Do excluded adolescents differ in* ***BehavioralControl_ASCS*** *from included adolescents?*

225 of the 235 excluded adolescents (95.7%) had reported a score on BehavioralControl_ASCS. These excluded adolescents scored similarly on BehavioralControl_ASCS as included adolescents.

**Conclusion**: Excluded YOUth adolescents have similarly educated parents, but are from lower income families than included adolescents; while scoring similarly on SocialCompetence_CBCL and on BehavioralControl_ASCS.

[**[back to index]**](#Index)
